# Can heart rate variability be a bio-index of hope? A pilot study

**DOI:** 10.3389/fpsyt.2023.1119925

**Published:** 2023-03-21

**Authors:** Ying Wai Bryan Ho, Daniel Bressington, Mei Yi Tsang, Hok Hoi Pang, Yan Li, Wai Kit Wong

**Affiliations:** ^1^School of Nursing, The Hong Kong Polytechnic University, Hong Kong, Hong Kong SAR, China; ^2^College of Nursing and Midwifery, Charles Darwin University, Casuarina, NT, Australia; ^3^Department of Occupational Therapy, Castle Peak Hospital, Hong Kong, Hong Kong SAR, China; ^4^Hong Kong Psychological Services Center Limited, Hong Kong, Hong Kong SAR, China; ^5^School of Nursing, Tung Wah College, Hong Kong, Hong Kong SAR, China

**Keywords:** Heart rate variability, HRV, hope, psychological well being, positive psychology

## Abstract

**Background:**

Hope can affect the thinking habits, emotional regulations, and behaviors of individuals. Hope is considered as a positive trait by clinicians, who often assess the level of hope in psychological evaluations. Previous measurements of hope were largely based on self-reported questionnaires leading to the problem of subjectivity. Heart Rate Variability (HRV) is a bio index that is an objective, quick, cost effective, and non-invasive measurement. HRV has been used in the evaluation of physical health and some psychiatric conditions. However, it has not been tested for its potential to be a bio-index of the level of hope.

**Method:**

This pilot cross-sectional observational study aimed to examine the relationships between HRV and the level of hope among adult Chinese people in Hong Kong. Convenience sampling was used and 97 healthy participants were recruited. Their level of hope was measured by the Dispositional Hope Scale-Chinese (DHS-C), and their HRV was quantified by emWave Pro Plus, a reliable sensor of HRV. Spearman’s correlation coefficient analysis was performed on the HRV measurements and DHS-C.

**Results:**

The DHS-C’s overall mean score was 45.49. The mean scores of the subscale DHS-C (Agency) was 22.46, and the mean scores of DHS-C (Pathway) was 23.03. It was also revealed that there were significant, weak, and negative correlations between the level of hope and four out of ten HRV metrics. One HRV metric was found to have a significant, weak, and positive correlation with the level of hope.

**Conclusion:**

This study provided initial evidence to support the use of HRV as a bio-index of hope. Implications of the current study and recommendations for future research directions are discussed.

## 1. Introduction

Hope has long been considered as an important therapeutic factor in psychology, medicine and nursing. Without hope, humans struggle to survive during challenging times. Hope can affect the thinking habits, emotional regulations, and behaviors of individuals (1). Individuals who are hopeful tend to have more goal-oriented thinking because this thinking style is rewarded by positive consequences (2, 3). Hope may also affect the emotion regulation processes of individuals; Peh et al. (4) conducted a path analysis to investigate whether hope mediates the association between reappraisal and anxiety/depression in patients newly diagnosed with cancer (*N* = 144). The result of study indicated that a higher level of reappraisal was correlated with lower levels of negative emotions and hope also mediated the linkages between reappraisal and negative emotions. Hope can also affect the behaviors of individuals. Dixson (5) conducted a cluster analysis which yielded three clusters of hope (high, average, and low) in high school and college students (*N* = 852). Differences among hope clusters were examined across three domains of indicator variables—(engagement, disengagement, and motivation)—associated with success-oriented behavior in school. The study finally revealed that hope could cause individuals to engage in more success-oriented behaviors (5).

Hope may also influence how people strive to achieve goals. Snyder et al. (2) described two different types of hope: dispositional hope and state hope. Dispositional hope is a trait like characteristic of individuals, which supports individuals to feel constantly hopeful. Whereas, state hope referred to current goal directed thinking, which occurred in short periods of time (6). Dispositional hope contained two interrelated cognitive dimensions: agency and pathway. Agency was the determination and commitment of individuals to help them move to the directions of goals (7). Agency thinking was the perceived capability of and motivation for achieving a desired goal. Individuals, who were high in this dimension, tended to empower themselves by self-talk phrases like, “I can do this all day” or, “I am not going to be stopped” (8). Pathway was described as the perceived capacity of individuals to achieve their goals and set up different plans to overcome the obstacles (7). Pathways thinking referred to the act of evaluating the availability of different resources and methods to reach the desired goals (3). Individuals who were considered as high in the pathways dimension tended to be more decisive and confident about creating workable routes for attaining the desired goal (8). In summary, hope was found to be a positive trait which constantly empowers individuals to seek possible pathways to achieve their desired goals (7).

Despite the potential importance of hope in influencing behaviors, it’s measurement in research studies is challenging. Previous measurements of hope are largely based on self-reported questionnaires, which include different scale including but not limited to the Work Hope Scale (9), Social Hope Scale (10), Locus-of-Hope Scale (11), and Dispositional Hope Scale (2). However, these scales have several fundamental problems in their design. Firstly, there is a problem of central tendency bias, which referred to the avoidance of participants to give answers on both extremes of the Likert scale, and the participants tended to respond in the mid-point (12). The second problem with self-reported questionnaires is that respondents’ answers may be heavily influenced by social desirability. Finally, the participants may have misconceptions about the wordings of the questions, since they are perceiving the questionnaires subjectively, which may be different from the original meanings of words used by the designers (13). In addition, the researchers using these measures require training. They are expensive and by nature the self-completed measures lack objectivity as they rely purely on self-report. Whereas bio indexes are objective, quick, cost effective, and non-invasive measurements.

Using bio-indexes to facilitate the assessment process is not new. For example, hospital nurses or physicians frequently use pulse rate, breathing rate, and blood pressure to investigate if individuals are anxious. Apart from these traditionally used bio-indexes, Heart rate variability (HRV) is a relatively new biometric which measures the fluctuation of time intervals between consecutive heartbeats (14). The variability of the heartbeat determines the capacity to the heart of individuals to alter their level of functioning to manage the changing external environment (15).

Previous studies demonstrated that Heart Rate Variability (HRV) could be an indicator of some physical health problems such as myocardial infarction, sepsis, trauma, sleep apnea, chronic fatigue and cardio-respiratory illnesses (16). Laborde et al. (17) proposed the neural pathway involved in the regulation of the autonomic nervous system (ANS) supports adaptive responses to the environment, stressors, and social behaviors (18). The heart’s activities increase in order to support rapid mobilization of metabolic resources which are required to prepare for appropriate actions (e.g., fight or flight response) (19).

Recent research has also revealed that HRV could reflect different psychiatric conditions, such as anxiety disorder, depression, post-traumatic stress disorder, daily mental stress and prolonged work stress (20–26). HRV can also provide an indication of mental wellbeing such as having good life satisfaction, a high level of positive affect and a low level of negative affect (27, 28). In summary, individuals with different ANS activities could have different HRV patterns. Hence, HRV could be used as bio-indexes of different mental health conditions.

Another direction of research on the usage of HRV on mental health was to investigate the correlations of HRV with positive psychological traits (i.e., resilience). Some studies demonstrated that HRV could be the biomarker of resilience, which is usually defined as a trait of individuals that help them adapt and bounce back from traumatic events or adversities, as it reflects the ability of individuals to maintain a stable equilibrium during stressful events (29, 30). Although recent research indicated that HRV could be bio-indexes of resilience, the possibilities of using HRV as the bio-index of other similar positive psychological traits such as hope have not been studied. The purpose of the current pilot study aimed to fill this research gap, which explored the possible correlations between HRV metrics and hope. If significant correlations between HRV metrics and hope were identified, new bio-indexes for hope would be established. As such, HRV would be able to be considered an alternative objective method to measure hope, without the inherent potential limitations of relying solely on self-reported questionnaires.

## 2. Theoretical framework

Polyvagal theory, which was proposed by Porges (18), provided a tenet for this study. According to this theory, the vagus nerve is a brake that actively inhibits the effect of the sympathetic nervous system (SNS) on heart activity to ensure individuals remain calm and relaxed when at rest. However, when individuals encounter stressful situations this vagal brake is rapidly reduced. Once the brake is released, then the heart’s activities increase in order to support rapid mobilization of metabolic resources which are required to prepare for appropriate action (e.g., fight or flight response) (19). Hence, the polyvagal tone could affect the activities of the ANS and the cognitive reappraisal of events and emotion regulations are also linked with cardiac vagal function. Meanwhile, hope could affect the cognitive reappraisal of events and emotional regulation capacities of individuals. Therefore, with regards to Polyvagal theory, hope should be linked with cardiac vagal function, which regulates the activities of ANS. As a result, the individuals’ difference in the level of hope should have effects on the polyvagal tone as well as the ANS, which could be measured by HRV. Hence, people with different levels of hope should have different patterns of HRV metrics. Supplementary Figure 1 illustrates the above-mentioned relationship.

## 3. Research questions

An individual’s level of hope can affect their capacities of emotion regulation and cognitive reappraisal, which impacts ANS activities. Given that alternations of the activities of ANS are revealed by HRV (31), differences in the level of hope should be reflected by the patterns of HRV metrics among different individuals. Thus, the HRV metrics could serve as bio-indexes of hope. In fact, as revealed by the literature review, HRV has been found to be able to serve as a biomarker of resilience, a similar psychological trait to hope. Therefore, the current research addresses the following research questions:

RQ1. To what extent is there a correlational relationship between HRV and the level of hope? RQ2. What is the overall level of hope among Hong Kong Chinese Adults?

## 4. Materials and methods

### 4.1. Study design

This study used a cross-sectional observational survey design to examine the relationships between HRV and the level of hope among adult Chinese people in Hong Kong.

### 4.2. Ethical consideration

Ethical approval was obtained from the Institutional Review Board (IRB) of the California Southern University (the institute where the first author completed his doctoral degree) before recruitment and data collection. All participants provided written informed consent. Participants were aware that taking part was voluntary and that they could withdraw from the study at any point without penalty. No monetary incentive was given to the participants.

### 4.3. Study setting

The researchers sent out online invitation letters *via* email, Facebook, and Instant Messenger of Smartphone (WhatsApp’s and Line) to invite participants to join the study in November 2021 in Hong Kong. The online invitation letters contain information including the purpose and nature of the study, measures to protect their privacy, participant inclusion and exclusion criteria, locations, duration, and procedures of the data collection, their rights to participate and withdraw from the study, the potential risk, and benefits of participating in this study as well as the contact information of the researcher.

### 4.4. Recruitment and data collection

The interested participants were invited to sign-up for a data collection session and were asked to input their information related to the participant inclusion and exclusion criteria *via* a Google form and select a preferred timeslot for data collection. Only the applicants who met the inclusion criteria were included in the study. Participants received a confirmation letter of the arrangements for data collection. The letter also detailed several precaution measures because these activities may affect the HRV readings. This included advice that participants should refrain from heavy aerobic exercise or consuming coffee, tea or other caffeinated beverages at least 1 h before data collection. Participants were also informed that they should not eat a heavy meal for at least one and a half hours before the assessment. In addition, they should be non-smokers. Recruitment and data collection were conducted from November 2021 to December 2021.

Data were collected at a University nursing laboratory. On the day of the data collection, the researcher initially confirmed whether the participants had followed the precautions measures and explained the purpose and the procedure of the study. After obtaining their informed consent, the researcher measured the vital signs (blood pressure, heart rate, and temperature), height and weight. The researcher assessed if the vital signs and their Body Mass Indexes (BMI) were within the normal range (i.e., BMI 18.5–22.9) and requested the participants to confirm they had no major illnesses. After that, the participants were invited to use their smartphone to click a link, which lead them to Qualtrics (i.e., an online platform for questionnaire-based research). They were requested to complete the online DHS-C and provide their demographic data (age, gender, educational level, marital status, and income) *via* the online form. Data from the online based questionnaires were stored on Qualtrics, which required the researcher’s login ID and password to access.

After the participants completed the questionnaires, their HRV was assessed by the emWave Pro Plus device in a quiet and air-conditioned room (between 23–25 degrees Celsius), so that no other unnecessary external stimulus, such as background noise, could affect the HRV parameters. During the HRV assessments, the participants were advised to sit in a comfortable chair with a backrest without talking, falling asleep, crossing legs, and making unnecessary movements which might cause unwanted artifacts. In addition, they were advised to open their eyes, while avoiding reading or engaging in intense mental activity during the HRV assessment. The sensor of the device was attached to the earlobe of the participant and the researcher confirmed that the signals were captured and visualized by the computer. The HRV data collection lasted for 5 min. The HRV data was stored within the computer of the researcher. The computer, which was locked with login ID and password, was stored inside a secure locker. All data including the HRV data, and the online questionnaire data was transferred onto a physical hard drive, which was encrypted. The hard drive will be stored in a locker in the research office for 5 years. Following the usual practice in handling research data, the data of in the hard drive would be deleted after 5 years (32).

### 4.5. Participants

Convenience sampling was used in this study. Those whose age ranged from 18–65 years and were able to read and write Chinese were included in this study. Those who had major illnesses (such as cardiovascular, endocrine, neurological, and psychiatric disorders), obesity or a history of alcohol, tobacco or substance misuse were not eligible to participate, as these problems might serve as confounding variables that can influence the baseline HRV measurements.

### 4.6. Variables

The Chinese version of the Dispositional Hope Scale was used to quantify the level of hope among the participants. Ten HRV metrics [Very Low Frequency (VLF), Low Frequency (LF), High Frequency (HF), Total Power (TP), Normalized Coherence (NC), Mean Heat Rate Range (MHRR), Mean Heart Rate (MHR), Standard Deviation of the Normal-to-Normal sinus-initiated interbeat-intervals measured in milliseconds (SDNN), Root Mean Square of Successive Differences between normal heartbeats (RMSDD), and Mean Interbeat Interval (MIBI)] were assessed by emWave Pro Plus, a reliable device to quantify HRV by using Photoplethysmography (PPG) technology.

### 4.7. Measurements

#### 4.7.1. Dispositional Hope Scale–Chinese

The level of hope was measured by the Dispositional Hope Scale, Chinese version (DHS-C) (33). The original Dispositional Hope Scale (DHS) was developed based on Snyder’s Hope theory (2). It used 4-point Likert (i.e., ranging from 1 = definitely false, 2 = mostly false, 3 = mostly true, and 4 = definitely true) and consisted of 12-items, which contributed to the total score of the scale. The scale is comprised of two subscales: agency thinking (i.e., contained four items) and pathways thinking (i.e., contained four items). The remaining four items were used as filter items. Low total scores indicated a respondent’s low level of hope while high scores indicated the opposite. DHS is a well validated (validity: 0.71–0.84; reliability: 0.73–0.85) tool to assess dispositional hope among college students and patients. (2) The scale was translated and validated to Chinese as Dispositional Hope Scale, Chinese Version (DHS-C) by Sun et al. (33). It used 8-point Likert (i.e., 1 = definitely false to 8 = definitely true) with the same 12-items and two subscales as the original version. The DHS-C, which demonstrated good structural validity (i.e., Δχ2(1) = 9.04, *p* < 0.01; CFI = 0.95) with 2-factor model of hope, was reported to have good psychometric properties and be suitable to assess the level of hope among Chinese people (33). Permission to use this scale was obtained from the author before the start of this study.

#### 4.7.2. emWave pro plus

In this study, the metrics of heart rate variability (HRV) were captured by the emWave^®^ Pro Plus system (HeartMath LLC., Boulder Creek, CA, United States). This tool, which applies photoplethysmography (PPG) technology, was able to detect and analyze the blood pulse wave through the skin. The wave in the blood stream is captured by a sensor, which should be attached to the ear lobe of the subjects. The data is then sent to the computer (MacBook Pro, 13-inch, M1, 2020) with emWave Pro Plus software (version 3.10.0.11205) for analysis. The device can provide two major domain measures of HRV; a time domain measure and a frequencies domain measure (34). The time domain related HRV metrics include the Mean InterBeat Interval (MIBI) and the Mean Heart Rate Range (MHRR). These parameters provide clinical information for effective analysis of the alternations of the heart rates of individuals caused by the actions of the SNS and PNS (15, 34). The Standard Deviation of all normal Interbeat Interval (SDNN) is considered as the gold standard of cardiac risk and Root Mean Square of Successive Difference (RMSSD) is associated with higher risk of sudden unexplained death among patients with epilepsy (15). In addition to the HRV metrics, the emWave Pro Plus also measured the Mean Heat Rate (MHR) for analysis.

The measures related to the frequency domain offered valuable clinical information about the functioning of the ANS, including the TP, VLF, LF, HF, and NC. PPG technology was considered as a reliable and valid method of capturing and quantifying HRV (35). The emWave Pro Plus has been used extensively in published research involving HRV measurements (36–40).

### 4.8. Study size

As there was no previous study investigating the correlations between HRV and hope in a Chinese context, the expected correlation was taken from a relevant local study, which investigated the correlations between stress and HRV among Chinese people (41). In Low and McCraty’s (41) study, the research designers estimated the correlation r as 0.3. To achieve a power of 0.8, an estimated correlation r of 0.3, with a significance level 0.05, the total sample size needed for this study was 85 participants (42).

### 4.9. Data analysis

Data were analyzed by Statistical Package for Social Science version 23 (SPSS) software. Missing values analysis was done and no patterns in missing data were detected. Subsequently, pairwise deletion was used to deal with missing data. Frequency and percentages were used to describe demographic data variables: gender, age, marital status, educational level, and family income. The total score of the DHS-C was used to measure the overall level of hope of participants.

After establishing that data were normally distributed, Spearman’s correlation coefficient analysis was performed on HRV measurements (MIBI, SDNN, RMSSD, MHRR, MHR, NC, TP, VLF, LF, and HF) and the score of DHS-C in order to investigate the correlational relationships among these variables and answer RQ1: To what extent is there a correlational relationship between HRV and the level of hope. The significance or alpha level for these analyses was set at *p* < 0.05.

## 5. Results

### 5.1. Participants

Overall, 102 individuals expressed an interest to participate in the research. However, five did not attend the assigned day for data collection. Finally, 97 individuals met the inclusion criteria and did not have any conditions listed in the exclusion criteria. They signed the consent forms and participated in the study (Supplementary Figure 2).

More than half of participants (*n* = 53, 54.6%) were male and the age ranged from 18 to 30 (53.6%). Nearly two-thirds of participants were single (*n* = 66; 68%). Most participants possessed an education of at least of Bachelor level (34%; *n* = 33). Overall, 29.9% (*n* = 29) of the participants had a Master’s degree, and 6.2% (*n* = 6) were educated up to doctoral level. Most of the participants (35.1%; *n* = 34) reported that their family income was above $50,000 Hong Kong Dollars per year. The demographic characteristics of the participants are presented in Supplementary Table 1.

### 5.2. The overall level of hope among Chinese adults

Of the total 97 respondents, the DHS-C (Total)’s overall mean score was 45.49, ranging from 12 to 96, with the standard deviation of 5.350. The mean scores of the subscale DHS-C (Agency) was 22.46, ranging from 4 to 32, with the standard deviation of 2.909. The mean scores of DHS-C (Pathway) was 23.03, ranging from 4 to 32, with the standard deviation of 3.306. Details of the scores are summarized in Supplementary Table 2.

### 5.3. Correlational relationship between HRV and the DHS-C (total scale)

Referring to Supplementary Table 3, four HRV metrics were found to be significantly, negatively, and weakly correlated to the total scale of DHS-C, which includes HF (*r* = −0.286, *n* = 97, *p* = 0.004), SDNN (*r* = −0.224, *n* = 97, *p* = 0.027), MIBI (*r* = −0.227, *n* = 97, *p* = 0.025), and RMSSD (*r* = −0.214, *n* = 97, *p* = 0.035). Three HRV metrics showed trends toward significant and negative correlations with the total score of DHS*-C.* These include VLF (*r* = −0.187, *n* = 97, *p* = 0.067), TP (*r* = −0.189, *n* = 97, *p* = 0.067), and LF (*r* = −0.155, *n* = 97, *p* = 0.131). However, MHR was significantly and positively correlated with the total scale of DHS-C (*r* = −0.223, *n* = 97, *p* = 0.028). The research results provide initial evidence that HRV metrics (HF, SDNN, RMSSD, and MIBI) could be considered as bio-indexes of the level of hope among individuals.

### 5.4. Correlational relationship between HRV and the DHS-C-pathway

Referring to Supplementary Table 4, Spearman’s Correlation analysis was conducted to analyze the correlations between DHS-C-pathway and HRV metrics. Significant and weak correlational relationships between 3 of the HRV metrics and the DHS-C-Pathway were observed. There was a significant correlation between the DHS-C-Pathway and HF (*r* = −0.261, *n* = 97, *p* = 0.010). Another significant correlation was observed between DHS-C-Pathway and RMSSD (*r* = −0.226, *n* = 97, *p* = 0.026). MHR was also significantly correlated with DHS-C-Pathway (*r* = 0.200, *n* = 97, *p* = 0.050). In addition, MIBI was observed to have significant correlation with DHS-C-Pathway (*r* = −0.204, *n* = 97, *p* = 0.045). There appeared to be a trend toward a significant negative correlation between DHS-C-Pathway scores and VLF (*r* = −0.188, *n* = 97, *p* = 0.066). Another trend between DHS-C-Pathway scores and SDNN was observed (*r* = −0.195, *n* = 97, *p* = 0.056).

### 5.5. Correlational relationship between HRV and the DHS-C-agency

Referring to Supplementary Table 5, Spearman’s Correlation analysis was done to analyze the correlations between DHS-C-Agency and HRV metrics. Significant correlational relationships between 1 of the HRV metrics and the DHS-C-Agency were observed. There was a significant and weak correlation between the DHS-C-Agency and HF (*r* = −0.243, *n* = 97, *p* = 0.016). There appeared to be a trend toward a significant negative correlation between DHS-C-Agency scores and SDNN (*r* = −0.195, *n* = 97, *p* = 0.056). Another trend between DHS-C-Agency scores and MIBI was observed (*r* = −0.189, *n* = 97, *p* = 0.063). Finally, there was a trend between DHS-C-Agency scores and MHR (*r* = −0.187, *n* = 97, *p* = 0.067).

## 6. Discussion

This is the first study providing initial evidence to support the potential use of HRV metrics as bio-indexes of hope. The results indicate that there were significant, weak, and negative correlations between most of the HRV metrics (i.e., HF, SDNN, RMSSD, and MIBI) and the level of hope. There was also a trend toward significant negative correlations observed on VLF, TP, and LF of HRV metrics. Hence, individuals with a higher level of hope tended to have a lower level of HRV metrics including HF, SDNN, RMSSD, MIBI, VLF, TP, and LF. The results also revealed that the subscales (Pathway) tend to have more negative correlations with HRV indexes. This result indicates that an individual’s act of evaluating the availability of different resources and methods to reach the desired goals tend to have negative impacts on HRV indexes.

Previous studies revealed that individuals with a lower level of HRV metrics tend to have poor health status, such as immune dysfunction and inflammation, cardiovascular disease, and mortality [Dekker et al. (43); Fang et al. (44); Kemp and Quintana (45)]. In addition, the current study revealed that higher level of hope was positively correlated with higher level of MHR, which was measured at resting status in this study. Higher resting heart rate could be seen as an index of poorer cardiovascular health and cardiovascular disease such as hypertension (46). In other words, positive traits, such as hope, can bring positive mental health outcomes for individuals (47) but they might impose negative effects on physical health in the population being studied.

The phenomenon observed in the present study could be explained by Segerstrom’s (48) Engagement Hypothesis. Segerstrom (48) proposed that individuals with more positive traits (e.g., optimism) tended to have more active engagement to the stressful environment during periods of adversity. These individuals believed that the active engagement with stressors could lead to termination of their stressor by participating in more problem-solving activities. However, if the circumstances were too challenging or demanding, individuals with more positive traits (e.g., optimism) tended to have poorer immunity, because these traits resulted in individuals being continuously focused on the stressful situation in an attempt to resolve the issues (49). In contrast, if the individuals had fewer positive traits (e.g., more pessimistic), they tended to give up more easily, disengage, and avoid active engagement in coping against the adversities. Hence, their physiological functioning, such as level of immunity, was less affected by the extreme environment. One previous study of the relationship between HRV and resilience (a positive trait similar to hope), revealed that the score of resilience was also negatively correlated to HRV metrics (LF and HF) which echoes our findings (29). In addition, An et al. (29) study also reported that higher scores in resilience were significantly and positively correlated with more adaptive engagement to adverse situations, i.e., an individuals’ tendency to actively participate in more adaptive behaviors during a challenging time (29). In other words, individuals with more positive traits (e.g., hope, optimism, and resilience) could have greater engagement with stressors during extreme situations, resulting in higher levels of demand on physiological functioning. In contrast, participants with lower levels of hope tended to have less engagement with adverse situations, resulting in fewer demands on physiological functioning. Even though people with positive traits were more likely to handle a higher level of stress and were apparently not distressed by it, stress could be reflected by objective measurements like HRV. The negative associations between HRV metrics and hope observed in the current study may have been due to participants’ active engagement with the stressful environment during COVID-19. During this study, the research participants were residing in Hong Kong during the COVID-19 pandemic; threats of infections, mandatory quarantine, lockdowns, and layoffs were seriously affecting the metropolitan. Most of the Hong Kong citizens were suffering from a greater level of stress (i.e., increased by 28.3%) and anxiety (i.e., increased by 42.3%), and the depression symptoms and unhappiness have doubled since COVID-19 outbreak when compared to 2016 and 2017 (50). With that premise, the Engagement Hypothesis could be applied, because the environments became demanding and challenging as evidenced by the elevated stress among the Hong Kong people.

As indicated in the Engagement Hypothesis, individuals, who possessed more of these positive traits (e.g., hope, optimism, and resilience), tended to be more engaged in the stressful environments. This difference was because these individuals tended to believe that they could tackle the stressors. The more frequent exposure to stressors may have resulted in a higher level of stress and extra physiological costs, which were captured by the reduced HRV metrics. Hence, negative and significant correlations were observed between the HRV metrics and hope in the current study.

### 6.1. Study limitations

The current study is limited by several factors. Firstly, it used a cross-sectional design. The primary limitation of this kind of research is that the researchers are not able to demonstrate temporal relationships between the variables. This is because these variables are measured and exposed at the same time. Secondly, as this study did not receive any funding support, the researchers had limited resources necessitating the use of convenience sampling. The major disadvantage of this sampling method is that the result may not be generalized to the wider population.

### 6.2. Recommendations for further research

Future research in the area should aim to replicate this study using a representative sample (including diverse ethnic groups) to estimate the possibility of using HRV metrics as bio-indexes of other positive psychological traits (i.e., optimism, wisdom, personal mastery, perceived self-efficacy, coping, creativity, conscientiousness, and spirituality and religiosity). Future studies should also consider conducting the research after the COVID-19 pandemic has resolved to examine any changes in the direction of the correlational relationships between HRV metrics and hope. In addition, this study identified significant, but weak correlations between HRV parameters and hope scales, which provides initial evidence for supporting to consider HRV as a possible bio-index of hope. However, previous literature indicated that other similar traits (e.g., resilience) also produced similar findings (29). Therefore, the research results should be interpreted with caution. A larger study that includes a range of other potentially confounding variables is recommended to identify which traits (e.g., hope or resilience) contribute to the HRV indexes before using HRV indexes as complementary measurements of hope. On the other hand, this study revealed that individuals, who scored high in the DHS-C, tended to have lower HRV. Future study is also needed to investigate the potential mediating relationships between engagement with stressors and HRV.

## 7. Implications

The results of current study provide initial evidence to support the use of HRV as a bio-index of hope. If researchers are able to generate more evidence which supports the linkage between HRV and hope in subsequent larger studies, then clinicians should consider using HRV metrics to assess the level of hope of their clients during clinical practice or/and research, alongside the self-report questionnaire. This approach could increase the objectivity of psychological evaluations and result in more reliable research outcomes. Measuring HRV is also cost effective, efficient, non-invasive, and straightforward.

The second implication for professional practice was that it alleviated the problem of assessment and re-assessment period of questionnaire-based clinical evaluation on the level of hope. In many questionnaire-based psychometric tests, time intervals between questionnaire administrations are required. Some researchers suggest that this time interval should range from 2 days to 2 weeks (51). Therefore, frontline psychologists had to wait until the next session to review the progress of their intervention. This problem also applied to the measurements of hope as they traditionally rely on questionnaire-based assessment. As the results indicated that HRV metrics have the potential to be a bio-index of this trait, the frontline psychologists could use the HRV metrics to evaluate and re-evaluate the level of hope within the same session. This change of practice provided a more direct and instant way for the frontline psychologists to review the effectiveness of their treatment. However, this second implication is subjected to the results of future study.

## Data availability statement

The raw data supporting the conclusions of this article will be made available by the authors, without undue reservation.

## Ethics statement

The studies involving human participants were reviewed and approved by the California Southern University. The patients/participants provided their written informed consent to participate in this study.

## Author contributions

YH and WW contributed to the conception and design of the study. YH and MT organized the database. YH performed the statistical analysis. YH, WW, and DB wrote the first draft of the manuscript. YH, YL, MT, HP, DB, and WW wrote sections of the manuscript. All authors contributed to the manuscript revision, read, and approved the submitted version.
