# Supplementary material for: Can heart rate variability be a bio-index of hope? A pilot study
Source: Front Psychiatry. 2023 Mar 21;14:1119925. doi: 10.3389/fpsyt.2023.1119925 (PMC10070701; doi:10.3389/fpsyt.2023.1119925)
Supplement: Supplementary file 1 [file Data_Sheet_1.docx]

Supplementary Material

Can Heart Rate Variability Be an Bio-index of Hope? A Pilot Study

# Ying Wai Bryan HO^1^*, Daniel BRESSINGTON^2^, Mei Yi TSANG^3^, Hok Hoi PANG^4^, Yan LI^1^ , Wai Kit WONG^5^*

# ^1^School of Nursing, The Hong Kong Polytechnic University, Hong Kong, China

# ^2^College of Nursing and Midwifery, Charles Darwin University, Casuarina, Australia

# ^3^Department of Occupational Therapy, Castle Peak Hospital, Hong Kong, China

# ^4^Hong Kong Psychological Services Center Limited, Hong Kong, China

# ^5^School of Nursing, Tung Wai College, Hong Kong, China

# * Correspondence:

# Corresponding Author: Ying Wai Bryan HO [ywbho@polyu.edu.hk](mailto:ywbho@polyu.edu.hk)

# Corresponding Author: Wai Kit WONG [Kelvinwong@twc.edu.hk](mailto:Kelvinwong@twc.edu.hk)

# Supplementary Figures and Tables

For more information on Supplementary Material and for details on the different file types accepted, please see [here](https://www.frontiersin.org/guidelines/author-guidelines#supplementary-material).

**
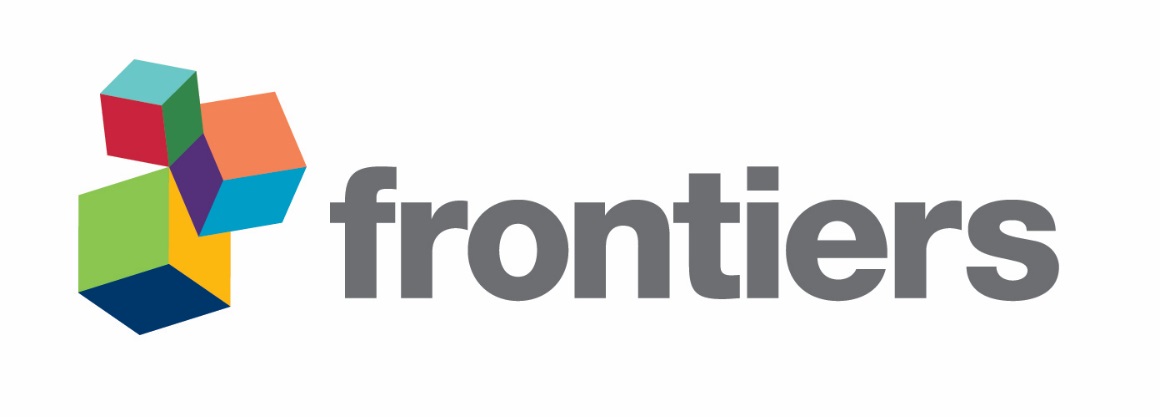
**

## Supplementary Figure 1. Evaluating the Level of Hope by Measuring HRV

Influence

Influence

Influence

Hope (Trait)

Emotion regulation & Cognitive Appraisal

Cardiac vagal function

ANS (measured by HRV)

## Supplementary Figure 2. The Recruitment of Samples

Participants expressed interest (n=120)

Excluded (n=5)

-Did not attend on the assessment day

Participated the study

(n=97)

Recruitment process

## Supplementary Table 1: Demographic Data of the Participants

| Total sample (n=97) |  |  | N | Percentage (%) | |
| --- | --- | --- | --- | --- | --- |
| Gender |  |  |  | |  |
|  | Male |  | 53 | | 54.6 |
|  | Female |  | 44 | | 45.4 |
| Age |  |  |  | |  |
|  | 18-30 |  | 52 | | 53.6 |
|  | 31-40 |  | 30 | | 30.9 |
|  | 41-50 |  | 9 | | 9.3 |
|  | 51-60 |  | 6 | | 6.2 |
| Marital Status |  |  |  | |  |
|  | Single |  | 66 | | 68 |
|  | Married |  | 28 | | 28.9 |
|  | Divorced |  | 2 | | 2.1 |
|  | Separated |  | 1 | | 1 |
| Education level |  |  |  | |  |
|  | Secondary |  | 16 | | 16.5 |
|  | Diploma/Certificate |  | 10 | | 10.3 |
|  | Associate Degree  /higher diploma |  | 3 | | 3.1 |
|  | Bachelor |  | 33 | | 34 |
|  | Master |  | 29 | | 29.9 |
|  | PhD/  Doctorate degree |  | 6 | | 6.2 |
| Family Income |  |  |  | |  |
|  | None |  | 2 | | 2.1 |
|  | Below $5000 |  | 7 | | 7.2 |
|  | $5001-$10000 |  | 2 | | 2.1 |
|  | $10001-$20000 |  | 15 | | 15.5 |
|  | $20001-$30000 |  | 20 | | 20.6 |
|  | $30001-$40000 |  | 10 | | 10.3 |
|  | $40001-$50000 |  | 7 | | 7.2 |
|  | Above $50000 |  | 34 | | 35.1 |

## Supplementary Table 2: Overall Level of Hope among the Participants

| Total sample  (n=97) | Range of Scale | N | Minimum | Maximum | Mean | Std. Deviation |
| --- | --- | --- | --- | --- | --- | --- |
| DHS-C (Total)  DHS-C (Agency)  DHS-C (Pathway) | 8-64  4-32  4-32 | 97  97  97 | 30  16  12 | 59  29  32 | 45.49  22.46  23.03 | 5.350  2.909  3.036 |

## Supplementary Table 3: The Correlational Relationship between DHS-C-Toal Scale & HRV Metrics

|  |  | VLF | LF | HF | TP | NC | MHRR | MHR | SDNN | RMSSD | MIBI |
| --- | --- | --- | --- | --- | --- | --- | --- | --- | --- | --- | --- |
| DHS-C | Spearman Correlation | -.187 | -.155 | -.286** | -.187 | .067 | -.280 | .223* | -.224* | -.214* | -.227* |
|  | Sig. (2-tailed) | .067 | .131 | .004 | .067 | .511 | .787 | .028 | .027 | .035 | .025 |

## Supplementary Table 4: The Correlational Relationship between DHS-C (Pathway) & HRV Metrics

|  |  | VLF | LF | HF | TP | NC | MHRR | MHR | SDNN | RMSSD | MIBI |
| --- | --- | --- | --- | --- | --- | --- | --- | --- | --- | --- | --- |
| DHS-C | Spearmen Correlation | -.188 | -.105 | -.261** | -.138 | .082 | -.037 | .200* | -.195 | -.226* | -.204* |
|  | Sig. (2-tailed) | .066 | .304 | .010 | .178 | .425 | .720 | .050 | .056 | .026 | .045 |

## Supplementary Table 5: The Correlational Relationship between DHS-C (Agency) & HRV Metrics

|  |  | VLF | LF | HF | TP | NC | MHRR | MHR | SDNN | RMSSD | MIBI |
| --- | --- | --- | --- | --- | --- | --- | --- | --- | --- | --- | --- |
| Hope (Agency) | Spearmen Correlation | -.143 | -.147 | -.243* | -.170 | .047 | -.019 | .187 | -.195 | -.158 | -.189 |
|  | Sig. (2-tailed) | .167 | .151 | .016 | .097 | .647 | .852 | .067 | .056 | .122 | .063 |
